# The role of flavor and fragrance chemicals in TRPA1 (transient receptor potential cation channel, member A1) activity associated with allergies

**DOI:** 10.1186/s13223-015-0074-0

**Published:** 2015-03-16

**Authors:** Satoru Mihara, Takayuki Shibamoto

**Affiliations:** 2-10-12 Nishinippori, Arakawa-ku, Tokyo, 116-0013 Japan; Department of Environmental Toxicology, University of California Davis, Davis, CA 95616 USA

**Keywords:** Flavor and fragrance chemicals, Allergy, TRPA1, Isothiocyanates, Bradykinin receptors

## Abstract

TRPA1 has been proposed to be associated with diverse sensory allergic reactions, including thermal (cold) nociception, hearing and allergic inflammatory conditions. Some naturally occurring compounds are known to activate TRPA1 by forming a Michael addition product with a cysteine residue of TRPA1 through covalent protein modification and, in consequence, to cause allergic reactions. The anti-allergic property of TRPA1 agonists may be due to the activation and subsequent desensitization of TRPA1 expressed in sensory neurons. In this review, naturally occurring TRPA1 antagonists, such as camphor, 1,8-cineole, menthol, borneol, fenchyl alcohol and 2-methylisoborneol, and TRPA1 agonists, including thymol, carvacrol, 1’*S*-1’- acetoxychavicol acetate, cinnamaldehyde, α-*n*-hexyl cinnamic aldehyde and thymoquinone as well as isothiocyanates and sulfides are discussed.

## Background

Allergies have been known as hypersensitivity disorders of the immune system since the beginning of the 19th century, and the concept of hay fever was described around same period. Later, it was proposed that allergic symptoms, such as asthma, were triggered by certain chemicals, in particular, naturally occurring ones [1]. For example, leukotrienes derived from arachidonic acid were hypothesized to play an important role in asthma [2]. Through studies conducted over the past decade, the association between immunogenic and neurogenic mechanisms in airway inflammation has been recognized [3,4]. It is also known that neuronal activation causes pain and irritation; neurogenic inflammation; mucus secretion; and reflex responses such as coughing, sneezing and bronchoconstriction.

Some of the agonists of transient receptor potential cation channel subfamily V member 1 (TRPV1) and transient receptor potential cation channel, member A1 (TRPA1) are reportedly potent tussive agents [3].

**Figure 1:**
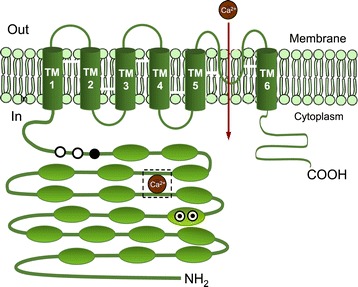
**Structure and function of one TRPA1 subunit.**

Figure 1 shows the structure of one TRPA1 subunit. TRPA1, which is a Ca^2+^ permeable non-selective cation channel, functions to depolarize the plasma membrane and influx Ca^2+^ [5]. The TRPA1 channel is a target of the mediators that promote inflammatory pain in the nervous system [6,7]. TRPA1 receptor agonists are chemicals that bind to TRPA1 receptors and activate the receptors to produce biological responses. Whereas TRPA1 receptor agonists cause actions, antagonists block the actions of the agonists. There are ankyrin repeat motifs in the intracellular N-terminal moiety of TRPA1. These moieties possess cysteine and lysine residues, which are essential for activation by reactive agonists. Also, a partial EF-hand domain, which is one of the motifs of a second structure of a protein, is associated with calcium-dependent gating [8,9].

A functional channel consists of 4 identical TRPA1 subunits. A subunit has six transmembrane domains, TM1 - TM6 along with a long cytoplasmic N-terminal domain. Ovals indicate ankyrin repeat domains. Cysteine residues of TRPA1 are marked with ⨀ for mouse, ○ for human and ● for mouse and human. They were essential for their covalent activation [10,11].

**Figure 2:**
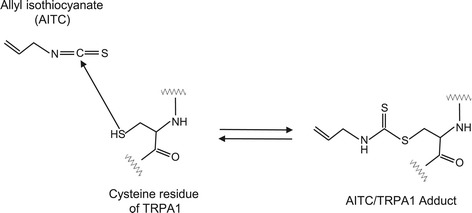
**Proposed reaction pathway of the activation of TRPA1 by a typical agonist, allyl isothiocyanates (AITC).**

TRPA1 is activated by various noxious stimuli, including cold temperatures, pungent natural-compounds, and environmental irritants [10,12]. Many TRPA1 agonists, which are thiol reactive compounds, activate TRPA1 *via* covalent modification of cysteine moieties within the cytoplasmic N terminus of the channel [11]. Figure 2 shows the proposed reaction pathway of the activation of TRPA1 by a typical agonist, allyl isothiocyanate (AITC). The figure is a modification of a previous report [11]. The EC_50_—half maximal (50%) effective concentration—values for the activation of TRPA1 by AITC vary among reports from as little as 0.6 μM [13], to 1.47 μM [14] and 3–34 μM [8]. TRP family members share this capacity as a polymodal signal detector, and these function to combine information from many physiological sources [11,15-17].

**Figure 3:**
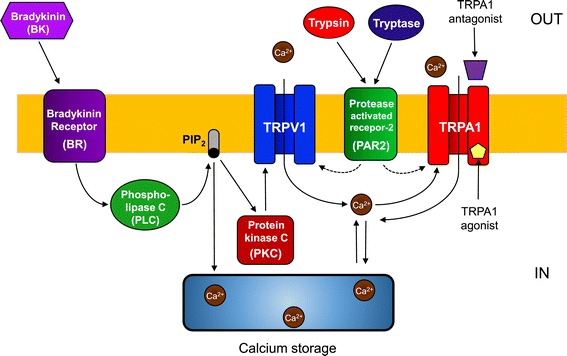
**Model depicting functional interactions in Bradykinin Receptors (BK), protease-activated receptors 2 (PAR2), and TRPA1 and TRPV1.**

Figure 3 shows a model depicting the functional interactions of bradykinin receptors (BK), protease-activated receptor 2 (PAR2), and TRPA1 and TRPV1. The figure is constructed on the basis of previous reports [7,8,18]. The hydrolysis of phosphatidylinositol 4,5-bis-phosphate (PIP_2_) and the intracellular Ca^2+^ release are phospholipase C (PLC)-dependent mechanisms, which activate TRPA1 downstream of inflammatory receptors [8]. Proinflammatory agents trypsin and tryptase are known to cleave to and to activate PAR2, which causes neurogenic inflammation by expressing on sensory nerves [19].

Genetic ablation of TRPA1 causes various biological phenomena, including inhibition of allergen-induced leukocyte infiltration in the airways, reduction of cytokine and mucus production, and significant disappearance of airway hyperreactivity to contractile stimuli. In addition, mouse model studies indicate that a TRPA1 antagonist inhibits chemical effects, such as thermal inflammation and mechanical hyperalgesia, neuropathic pain, and reduction of acute airway responses to chemical exposures [3]. This evidence indicates that TRPA1 is a crucial integrator of interactions between the immune and nervous systems that induces asthmatic inflammation in the airways following an inhaled allergen challenge [3,20]. Pharmacological desensitization of receptors is a basic mechanism of regulation of this kind of assault on neuronal systems [21]. TRPA1 is desensitized by its homologous agonists, such as allyl isothiocyanate (TRPA1 agonist) through the Ca^2+^-independent pathway and heterologous agonists, such as capsaicin (TRPV1 agonist), *via* the Ca^2+^-dependent pathway in the sensory neurons [5,21].

In this review, the roles of naturally occurring flavor and fragrance chemicals in TRPA1 activity associated with allergic disorders, such as asthma, eczema (atopic dermatitis) and allergic rhinitis are discussed along with the rationale for the use of TRPA1 as an anti-allergic target.

### TRPA1 Antagonists

**Figure 4:**
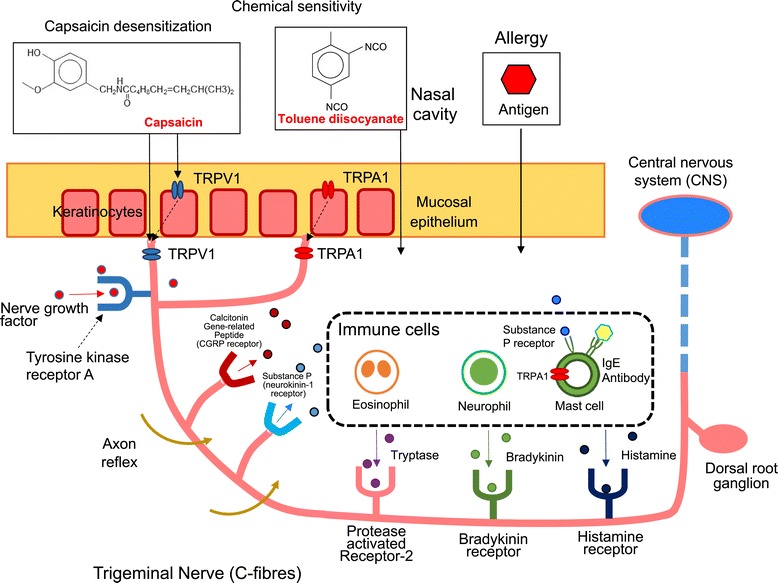
**Schematic diagram of nasal allergy-like symptoms induced by toluene diisocyanate (TDI) in rats.**

**Figure 5:**
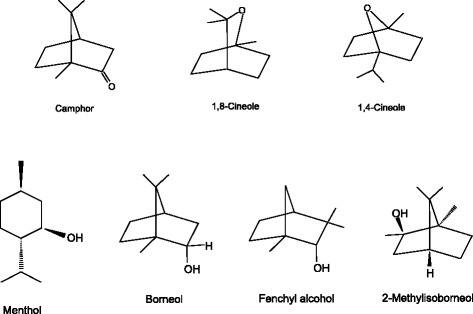
**Structures of TRPA1 antagonists discussed in this review.**

Figure 4 shows a schematic diagram of nasal allergy-like symptoms induced by toluene diisocyanate (TDI) in rats. The figure is based on previously reported diagrams [7,22]. The early phase of type I allergic reaction occurs when inflammatory mediators are released by environmental proteins (antigens) binding to IgE antibodies on the mast cells. The inflammatory reactions caused by environmental exposures are closely associated with allergy and chemical sensitivity, which are similar in the condition of clinical manifestations. When low molecular weight chemicals bind to chemoreceptors on sensory nerve C-fibers, inflammatory mediators are formed in the case of chemical sensitivity [7]. Although either TRPV1 or TRPA1 activation causes neurogenic airway inflammation, an additional inflammatory response which is not neurogenic is solely orchestrated by TRPA1 activation, suggesting that non-neuronal TRPA1 in the airways likely contributes to inflammatory airway diseases. Figure 5 shows the structures of TRPA1 antagonists discussed in this review.

### Camphor (1,7,7-trimethylbicyclo [2.2.1]heptan-2-one)

Camphor is a bicyclic monoterpene with a 204°C boiling point. It has a warm-minty fragrance [23]. It is present in natural plants, such as camphor laurel trees grown in Asia and has been used for artificial mint flavors and for some medicinal purposes, including as a nasal decongestant and cough suppressant as well as a skin treatment because of its antipruritic, analgesic and counterirritant properties [24-26]. One study found that when camphor was applied to nasal airways of guinea pigs, a cough induced by citric acid was suppressed [27]. There have been various reports on the biological activities of camphor toward TRPs. For example, camphor activated TRPV3 and heterologously expressed TRPV1, although its activity was somewhat less than that of capsaicin, the analgesic activity of which is also associated with TRPV1 desensitization. On the other hand, it was observed that camphor desensitized TRPV1 more quickly and perfectly than capsaicin [24,28]. The exposure to vapor phase camphor attenuated nasal symptoms (sneezing and nasal rubbing) induced by toluene diisocyanate (TDI) through the suppression of the production of neuropeptides, such as substance P (SP), calcitonin gene related peptide (CGRP) and nerve growth factor (NGF) in rats. These phenomena suggest the anti-allergic activities induced by camphor are due to the desensitization of TRPV1 and the blockage of TRPA1 [24,29].

### 1,8-Cineole (1,3,3-trimethyl-2-oxabicyclo[2.2.2]octane)

1,8-Cineole is a bicyclic mono-terpenoid colorless liquid. It has a fresh, diffusive camphoraceous-cool odor and is widely used for its refreshing effect in compounding perfumes with herbaceous type fragrances [23]. High levels of 1,8-cineole are present in various essential oils, including eucalyptus [30], laurel leaf [31], ravensara [32,33], cardamom [34], *Alpinia calcarata* Rosc [35,36] and *Nepeta pogonosperma* Jamzad et Assadi [37]. There are many reports on the biological activities of 1,8-cineole-rich essential oils, such as their antimicrobial [38,39], antioxidant [40], acaricidal [41], anticancer [42], larvicidal [43] and antinociceptive [44] effects. Consequently, many biological activities of 1,8-cineole itself have been reported, including its antimicrobial [45], antioxidant [46], anti-inflammatory [47], antiviral [48], anti-cancer [49] and antibacterial [50] properties. In addition, one recent study demonstrated that 1,8-cineole was a rare natural antagonist of human TRPA1 (hTRPA1) [51]. Further, 1,8-cineole has been shown to inhibit homologous passive cutaneous anaphylaxis (PCA) mediated by IgE antibody in guinea pigs and to have suppressed antigen-induced histamine release from rat peritoneal mast cells [52].

Exposure to vapor phase 1,8-cineole has been observed to suppress nasal symptoms (sneezing and nasal rubbing) induced by TDI by suppressing the production of neuropeptides in rats, just as camphor did, suggesting that it too has anti-allergic, analgesic and anti-inflammatory effects due to the inhibition of TRPA1 [29,51]. It is interesting that hTRPA1 is inhibited by 1,8-cineole but activated by its isomer, 1,4-cineole [53].

### Menthol [(1*R*,2*S*,5*R*)-2-isopropyl-5-methylcyclohexanol]

Menthol is a monocyclic monoterpene alcohol with a boiling point of 212°C. It is a clear or white solid at room temperature. Menthol has a refreshing and diffusive odor with a sweet pungency as well a characteristic peppermint odor. It has been widely used in food and cosmetic products as a flavor and fragrance ingredient. Some products utilizing menthol include imitation peppermint flavors for ice creams, cookies, chewing gums, lotions and shaving creams [23]. There are eight possible stereoisomers; the (−)-menthol assigned 1*R*,2*S*,5*R* configuration is the one generally present in natural plants. It is present in mint (*Mentha arvensis*) [52].

A recently published comprehensive review article summarizes menthol’s biological activities, including its cooling effect and its analgesic, antifungal, antibacterial, antipruritic, anticarcinogenic, anti-inflammatory, antitussive, antiviral and fumigant/insecticidal effects as well as a possible role in slowing the progression of Alzheimer’s disease [54]. Biological activities of menthol associated with allergy were demonstrated as menthol-rich peppermint oil and menthol itself were seen to suppress passive cutaneous anaphylaxis reaction (PCA) mediated by IgE antibody in guinea pigs and menthol reduced antigen-induced histamine release from rat peritoneal mast cells [52]. Moreover, menthol exhibits biological effects on TRPA1, such as a bimodal action on mouse TRPA1 (mTRPA1). Menthol induced robust channel activation at submicromolar to low micromolar concentrations but reversible channel blocking at higher concentrations in mice [55,56]. Menthol also activates human TRPA1 (hTRPA1), but the same bimodal action has not been reported, and it has no effects on TRPA1 from non-mammalians [55]. Serine and threonine residues—predicted to be located in the inner side of a transmembrane domain 5 (TM5)—were found to play an important role in the sensitivity of mice as well as humans, toward menthol in both mammalian TRPA1 channels (refer to Figure 1). Of the three agonists discussed above, menthol exhibited the most effective results on cough suppression in guinea pigs treated with aerosolized citric acid. Camphor exhibited moderate activity, whereas 1,8-cineole exhibited none [57]. When TRPM8 agonists, (−)- and (+)-menthols were applied to the nose, allergic reactions (cough threshold, urge to cough and cumulative cough) improved considerably, suggesting that menthol isomers possess a strong anti-irritant effect [58].

### Borneol (*endo*-1,7,7-trimethyl-bicyclo[2.2.1]heptan-2-ol)

Borneol comes in the form of colorless or white lumps at room temperature that melt at 208°C. It exists as two enantiomers and its naturally occurring form is *d*-(+)-borneol. Borneol has a woody, somewhat minty odor and is used as a fragrance ingredient for perfumes and household products, such as room-fresheners [23]. It has been found in various plants, including the Mei Pian tree [59], yomogi [60] and ginger [61]. Some biological activities, such as anti-inflammatory and analgesic, of borneol have been reported [62]. Recently, borneol, camphor, 1,8-cineole and α-/β-thujone were demonstrated to exhibit anti-inflammatory activity against sage infusion in human gingival fibroblasts [63].

### Fenchyl alcohol [(1*R*,2*R*,4*S*)-1,3,3-trimethyl-2-norbornanol]

Fenchyl alcohol is an isomer of borneol. It is a colorless solid crystalline that melts at 48°C. It possesses a powerful and diffusive camphor-like fragrance and is used extensively in perfumes. It is also used in flavor compositions, such as strawberry and other berries [23]. Fenchyl alcohol is present as the second largest component (8.9%) after aciphyllene (66.4%) in the essential oil *Stachys tibetica*, which has been used as a folk medicine in Ladakh, India and Tibet for the treatment of psychiatric disorders [64]. In contrast, fenchyl alcohol is also reported as an off-flavor compound formed microbially in apple juice [65].

### 2-Methylisoborneol (1,2,7,7-tetramethylbicyclo[2.2.1]heptan-2-ol)

2-Methylisoborneol is a derivate of borneol with a boiling point of 208.7°C. It is present in blue-green algae found in saline lakes in South Western Manitoba, Canada [66]. 2-Methylisoborneol has a unique strong musty or earthy odor and is associated with negative assessments of drinking water when present [67]. It is also reported in the essential oil of turmeric leaves (*Curcuma longa* L. *Kasur*) grown in Pakistan [68] and in the tea tree (*Melaleuca alternifolia*, Myrtaceae) grown in Australia [69]. An essential oil of the tea tree has been used in artificial fragrances for cosmetic products and also for treatment of infections, suggesting that its components exhibit biological activity [69].

Among the antagonists discussed in this section, borneol, 2-methylisoborneol and fenchyl alcohol had stronger inhibitory effects on hTRPA1 than camphor and 1,8-cineole. It is proposed that the S873, T874 and Y812 residues of hTRPA1 contributed to the inhibitory effects by interacting with a hydroxyl group on a hexyl ring [53].

### TRPA1 Agonists

**Figure 6:**
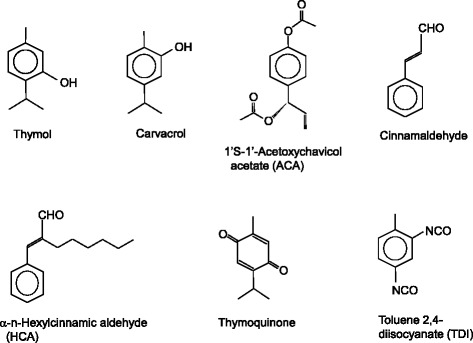
**Structures of aromatic TRPA1 agonists discussed in the present review.**

**Figure 7:**
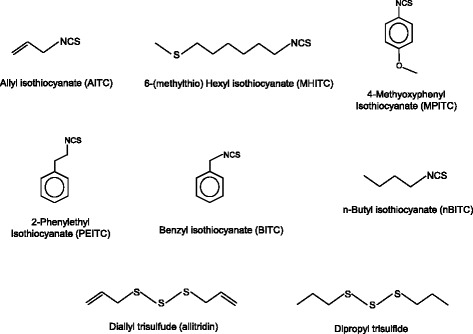
**Structures of nitrogen (isocyanate) or sulfur (sulfides) and nitrogen/sulfur (isothiocyanates) containing TRPA1 agonists discussed in the present review.**

Figure 6 shows structures of the aromatic TRPA1 agonists discussed in the present review. Figure 7 shows structures of the nitrogen (isocyanate) or sulfur (sulfides) and nitrogen/sulfur (isothiocyanates) containing TRPA1 agonists discussed in the present review.

### Thymol (2-isopropyl-5-methylphenol)

Thymol is a naturally occurring monoterpene aromatic alcohol and translucent crystal with a boiling point of 232°C [23]. It is the major component of thyme essential oil [70]. Thymol has a powerful, sweet-medicinal, herbaceous, and warm odor of moderate tenacity and its taste is pleasantly sweet-medicinal and herbaceous-spicy. Consequently, thymol has been used widely in flavor compositions for many products, such as toothpaste, cough drops, mouth-washes, gargles, and chewing gums [23].

Thymol is a well-known naturally occurring chemical with strong biological activities, including antibacterial [71], larvicidal [72], anti-inflammatory [73], nematicidal [74], acaricide [75], antifungal [76] and antioxidant [40] activities. When TDI-sensitized rats were exposed to vapor phase thymol, various biological effects, including sneeze suppression, inhibition of an increase of calcitonin gene-related peptide, and appearance of nerve growth factor in nasal lavage, were observed [77]. Nasal application of thymol suppressed the nasal problems, such as cough, both in guinea pigs and human volunteers [27,78].

Thymol activated a dose-dependent membrane potential response and intracellular calcium increase in hTRPA1-expressing HEK293 cells. Consequently, activation of hTRPA1 by thymol was observed [70,79]. On the other hand, once hTRPA1 was activated by thymol, further exposure to thymol desensitized the activated hTRPA1. This response by thymol was inhibited by the hTRPA1 antagonist camphor [79]. The anti-allergic property of thymol (TRPA1 agonists) may be due to the down-regulation (desensitization) of TRPA1 expressed in sensory neurons.

### Carvacrol (5-isopropyl-2-methylphenol)

Carvacrol, a colorless liquid with a boiling point of 237°C, is an isomer of thymol. It is a major component of the essential oils of oregano, thyme, and marjoram [80]. Carvacrol possesses phenolic herbaceous odor with a spicy undertone. The odor of carvacrol lacks sweetness compared with that of thymol. It has been used for household fragrances in products such as soap, air-freshener, shampoo, and mouthwash [23]. Carvacrol has quite similar biological activities to those of thymol, including antioxidant, antimicrobial and anti-inflammatory activities, and co-exists with thymol in the essential oils of thyme and oregano [70,76,81]. Carvacrol activates and then rapidly desensitizes TRPA1 [70]. For example, it reduced paw edema induced by histamine, dextran, and substance P, respectively, in mice [82].

### 1’*S*-1’-Acetoxychavicol acetate ([4-[(1*S*)-1-acetyloxyprop-2-enyl]phenyl] acetate)

1’ *S* -1’-Acetoxychavicol acetate (ACA) is present in various medicinal plants, such as ginger and *Alpinia* species grown in Malaysia and Thailand [83]. It possesses a pungent taste [84].

ACA is not widely used for fragrance composition, but it has been used in various medicinal treatments because of its biological activities, including anti-inflammatory, antiallergic, antifungal, antidiabetic, antibacterial, anticancer, and antioxidant [85] activities. In particular, ACA activities in cancer prevention, such as in the cases of breast [86], oral [87] and skin [88] cancers, has been reported.

There are many studies on the relationship of ACA to allergic reactions. Even though ACA did not activate TRPV1-expressing human embryonic kidney (HEK) cells, it strongly activated TRPA1- expressing HEK cells. The EC_50_ value of ACA for hTRPA1 (0.16 μM) was 3.8-fold lower than that of a typical TRPA1 agonist, allyl isothiocyanate (0.60 μM) [13]. The release of β-hexosaminidase, which is a marker of antigen-IgE-mediated degranulation in RBL-2H3 cells, was inhibited by ACA. In addition, ACA exhibited various biological activities, including inhibition of the ear passive cutaneous anaphylaxis reactions in mice, antigen-IgE-mediated TNF-alpha and IL-4 production associated with the late phase of type I allergic reactions [89], the reduction of white blood cell infiltration and IgE level in the lungs of mice administered OVA, the suppression of histopathological changes, and inhibiting expression of the various cytokines. Consequently, ACA is proposed to be an antiasthmatic drug candidate because asthmatic reactions are mediated by various immune and inflammatory pathways [90].

### Cinnamaldehyde [(*E*)-3-phenylprop-2-enal]

Cinnamaldehyde comprises over 90% of cinnamon essential oil, which has been used for various medicinal purposes, including as a styptic, an emmenagogue, a tonic for the liver, and to reduce inflammation, vomiting, and abdominal pains [91]. Some domestic medicines prepared from cinnamon plants have been used to treat diseases, including nasal allergies [92] and lung inflammation [93]. The herbal medicine, called “Kampo “or Chinese medicine, “Shoseiryuto” is prepared from eight plants, including Cinnamomi Cortex (cinnamon); it has been widely used in Japan [93].

Cinnamaldehyde is a pale yellow viscous liquid with a boiling point of 248°C. It possesses a warm-spicy-balsamic odor as well as a sweet and warm-spicy taste [23].

It has been used widely as a fragrance ingredient in many products, including cosmetics, shampoos, soaps, and perfumes as well as in household cleaners and detergents [94]. Above all, cinnamaldehyde has been used widely in flavor compositions, such as cinnamon, cola, mint, and cherry, because of its unique taste [23]. A comprehensive review article on the biological activities of cinnamaldehyde, including its neurotoxicity, mutagenicity/anti-mutagenicity, cytotoxicity, and carcinogenicity, is available [94]. The activities of cinnamaldehyde associated with TRPA1 have also been reported. Saturating activation by cinnamaldehyde blocked the effectiveness of TRPA1 channels [95]. Cinnamaldehyde reportedly activated cloned human TRPA1 channels in HEK293 cells as well as vagal sensory nerves in murine, guinea pig, and human tissues. It also induced reproducible tussive responses in both guinea pigs and humans [96]. It is proposed that TRPA1 undergoes pharmacological desensitization through agonist-dependent multiple cellular pathways, which are regulated by TRPV1 [21]. There is clear evidence that oral administration of cinnamaldehyde decreased oral irritation in humans [97].

### α-*n*-Hexyl cinnamic aldehyde [(2*E*)-2-benzylideneoctanal]

**Figure 8:**
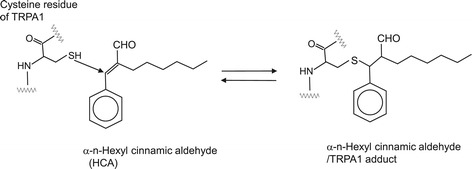
**Proposed reaction mechanism of HCA/TRPA1 adduct formation.**

α-*n*-Hexyl cinnamic aldehyde (HCA) is a pale yellow liquid with a boiling point of 308°C. HCA has not been found in natural plants but can be synthesized by condensation of benzaldehyde and octanal under basic conditions. It has a unique sweet-herbaceous/floral odor and is heavily used in floral perfume formulations, such as jasmine, gardenia, tuberose, and magnolia [23]. When rats were exposed to volatile HCA, their TDI-induced nasal symptoms (sneezing and nasal rubbing) were suppressed. These effects were associated with the suppression of the production of neuropeptides like SP, CGRP and NGF [98]. Figure 8 shows a proposed reaction mechanism of HCA/TRPA1 adduct formation. This reaction occurs through activation of TRPA1 by α,β − unsaturated aldehydes, such as HCA, subsequent to which adducts are formed upon Michael addition reaction [8]. HCA also has been demonstrated to activate TRPA1 *via* covalent protein modification.

### Thymoquinone (2-isopropyl-5-methylbenzo-1,4-quinone)

**Figure 9:**
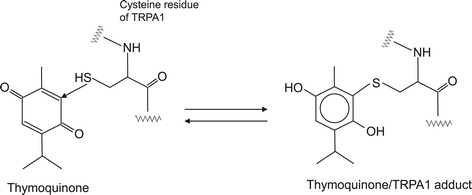
**Proposed formation mechanism of thymoquinone/TRPA1 adduct** ***via*** **Michael addition.**

Thymoquinone is a monoterpene diketone with a boiling point of 230–232°C. It is the major monoterpene present in the essential oil of *Nigella sativa* L seeds, which has been used as a folk medicine for various diseases, such as eczema, asthma, bronchitis, and inflammation [73,99,100]. This thymoquinone containing plant demonstrates some biological activities, including anti-tumor [101], cytotoxic and immunopotentiating [102], anti-inflammatory [103,104] as well as respiratory stimulatory effects [105]. Figure 9 shows a proposed formation mechanism for the thymoquinone/TRPA1 adduct *via* the Michael addition. Quinones, including thymoquinone, react with cellular nucleophiles such as thiols or amines. Thymoquinone may activate TRPA1 through covalent protein modification [106,107]. As shown in Figure 1, the presence of several cysteine residues is necessary in order to activate TRPA1 by thymoquinone [11].

### Toluene 2,4-diisocyanate (2,4-diisocyanato-1-methylbenzene)

Toluene 2,4-diisocyanate (TDI) has not been reported in natural plants. However, a brief description of TDI is given because it has been commonly and widely used in model studies of allergies. Moreover, TDI appears many times in this review (see Figure 4). It is a powerful irritant to the mucosal membranes of the respiratory tracts, eyes, and skin [108] and causes various respiratory symptoms, such as coughing, rhinitis, and dyspnea as well as chest tightness, in people who work in factories associated with this chemical [109]. When TDI was administered intranasally to guinea pigs, nasal allergy-like symptoms (sneezing and watery rhinorrhea) were observed; but when the TRPV1 agonist, capsaicin, was administered before the treatment with TDI, those symptoms were not observed. Capsaicin desensitization also inhibits the formation of histamine by TDI in the nasal mucosa. These reports suggest that the antidromic impulses (Axon reflex) from capsaicin-sensitive nerves induce histamine in the nasal mucosa upon TDI stimulation and, consequently, nasal discharge and sneezing occurs in guinea pigs (refer to Figure 4 for the mechanism) [110]. The up-regulation of histamine H (1) receptor (HIR) and histidine decarboxylase (HDC) gene expressions were also induced by TDI [111].

The activation of TRPA1 triggers release of pro-inflammatory neuropeptides, such as substance P or CGRP, by elevating Ca^2+^ levels in neurons [8]. A subset of dissociated trigeminal sensory neurons from wild-type mice was activated by TDI, but the same subset from TRPA1-deficient mice was not activated by TDI. TDI caused a reduction in the breathing rate and respiratory sensory irritation in wild-type mice, but not in TRPA1-deficient mice. They also exhibited some sensory effects, such as nerve activation and airway irritation *via* the activation of the ion channel [109].

### Isothiocyanates

The chemicals called isothiocyanate contain the -NCS moiety. They have a sulfurous odor.

In particular, allyl isothiocyanate (AITC)—a colorless liquid with a boiling point of 148–154°C—and 6-(methylthio)hexyl isothiocyanate (MHITC) give the characteristic green tone and pungent odor to Wasabi (*Wasabia japonica* (Miquel) Matsumura) [112]. Wasabi extract has been used as a domestic medicine for many purposes, such as antimicrobial, deodorization and detoxification [113] treatments as well as to improve the atopic-dermatitis-like symptoms of HR-1 hairless mice [114]. One study reported that sulforaphane (4-methylsulfinyl butyl isothiocyanate)—found in broccoli sprouts with five other isothiocyanates—exhibited potent anti-*helicobacter* activity [115].

Exposure to vapor phase MHITC [116], 4-methoxyphenyl isothiocyanate (MPITC), 2-phenethyl isothiocyanate (PEITC), benzyl isothiocyanate (BITC) and *n*-butyl isothiocyanate (nBITC) (refer to Figure 7 for structures) inhibited nasal symptoms (sneezing and nasal rubbing) induced by TDI *via* suppression of the production of neuropeptides, such as SP, CGRP and NGF, in rats [117].

MHITC reportedly activated both mTRPA1 and hTRPA1, suggesting that these biological activities of isothiocyanates are due to TRPA1 activation [112]. TRPA1 is a cation channel and is co-expressed with the TRPV1 channel in primary sensory neurons. TRPV1-specific agonist capsaicin and a typical TRPA1 agonist, allyl isothiocyanate (AITC) reportedly exhibited functional cross-desensitization in various rat and human models. Capsaicin- and AITC-induced calcitonin gene-related peptide (CGRP) release was 50–60% inhibited by pretreatment, indicating that homologous and heterologous desensitization occurred [118]. As shown in Figure 2, isothiocyanates are membrane-permeable electrophiles that form adducts with thiols and primary amines, suggesting that covalent modification, rather than classical lock-and-key binding, accounts for their agonist properties [11]. The anti-allergic properties of isothiocyanates may be due to the down-regulation (desensitization) of TRPA1 expressed in sensory neurons [21].

### Diallyl trisulfide [3-(prop-2-enyltrisulfanyl)prop-1-ene]

**Figure 10:**
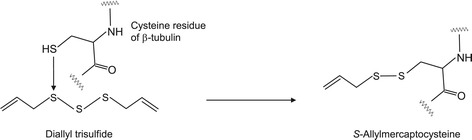
**Proposed formation pathway of** ***S*** **-allylmercaptocysteine from the reaction between diallyl trisulfide and a specific cysteine moiety of** **β** **-tubulin.**

Sulfide compounds are known to be present in garlic (*Allium sativum* L.). They are formed from sulfur-containing amino acids, such as cysteine and methionine, in garlic and are known to have various medicinal properties, including antibacterial, antithrombotic, cardiovascular, and anticarcinogenic activities [119-121]. Diallyl trisulfide, or allitridin, is a yellow liquid with an experimental boiling point of 251–262°C. It possesses a strong garlic odor and exhibits anticarcinogenic activity [122,123]. The EC_50_ values of diallyl trisulfide and allyl isothiocyanate (AITC) for hTRPA1 are 0.49 μM and 1.47 μM, respectively, suggesting that diallyl trisulfide is a more potent agonist than AITC, a typical hTRPA1 agonist [14]. Figure 10 shows the formation pathway of *S-*allylmercaptocysteine from a reaction between diallyl trisulfide and a specific cysteine moiety of β-tubulin, which is in the globular protein family. This reaction suggests that diallyl trisulfide is responsible, at least in part, for the anticarcinogenic effect of garlic [122].

### Dipropyl trisulfide [1-(propyltrisulfanyl)propane]

Dipropyl trisulfide has a typical sulfurous flavor and a boiling point of 256.8°C. It is a major component in the essential oil of leeks, *Allium porrum* L. (Alliaceae) and exhibits antimicrobial activity [124]. As in the case of camphor, 1,8-cineole and thymol, exposure to vapor phase dipropyl trisulfide attenuated nasal symptoms (sneezing and nasal rubbing) induced by TDI in rats, indicating that prolonged exposure to dipropyl trisulfide, which is a probable TRPA1 agonist, desensitized the nociceptive receptor TRPA1 (Figure 1) [125].

Studies using chemicals with strong irritant activity are limited to in vitro studies with cells or animal studies. Quantities of the irritant chemicals and study methods are strictly limited in human studies. For example, a Chinese herb medicine, cinnamon containing cinnamaldehyde, has been used to treat sinus allergies. Directions for use and dosage are established based on long term experience. However, there is almost no information derived from human clinical studies on the anti-allergic activities of fragrance chemicals.

## Conclusions

There are two groups of naturally occurring flavor and fragrance chemicals associated with TRPA1: one group comprises the antagonists, such as camphor, 1,8-cineole, menthol, borneol and fenchyl alcohol; and the other group is the agonists, such as thymol, carvacrol, 1’*S*-1’-acetoxychavicol acetate, cinnamaldehyde, thymoquinone and isothiocyanates. TRPA1 antagonists have anti-inflammatory and anti-allergic effects, possibly due to their TRPA1 blocking activity expressed in sensory neurons. On the other hand, activation and subsequent down-regulation of TRPA1 expressed in sensory neurons (desensitization) may be associated with the anti-allergic property of the TRPA1 agonists. Fragrance chemicals associated with TRPA1 are extremely important because they play a key role in allergic reactions. Therefore, investigation of how TRPA1 reacts in tandem with other chemicals is one way to elucidate various allergic mechanisms and to further efforts to improve drug treatments to prevent allergic reactions.
